# Mediterranean Diet Prior to Ischemic Stroke and Potential Circulating Mediators of Favorable Outcomes

**DOI:** 10.3390/nu16183218

**Published:** 2024-09-23

**Authors:** María Castañón-Apilánez, Carmen García-Cabo, Cristina Martin-Martin, Belén Prieto, Eva Cernuda-Morollón, Pablo Rodríguez-González, Daniela Pineda-Cevallos, Lorena Benavente, Sergio Calleja, Elena López-Cancio

**Affiliations:** 1 Department of Neurology, Hospital Universitario Central de Asturias (HUCA), 33011 Oviedo, Spain; 2 Instituto de Investigación Sanitaria del Principado de Asturias (ISPA), 33011 Oviedo, Spain; 3 Translational Immmunology, Instituto de Investigación Sanitaria del Principado de Asturias (ISPA), 33011 Oviedo, Spain; 4 Clinical Biochemistry Service, Laboratory of Medicine, Hospital Universitario Central de Asturias (HUCA), 33011 Oviedo, Spain; 5 Department of Physical and Analytical Chemistry, University of Oviedo, 33006 Oviedo, Spain; 6 Department of Funcional Biology, Universidad de Oviedo, 33003 Oviedo, Spain

**Keywords:** Mediterranean diet, resistin, progenitor endothelial cells, neuroprotection, insulin resistance

## Abstract

**Background/Objectives**. A Mediterranean diet (MD) has been associated with neuroprotective effects. We aimed to assess the MD’s association with stroke prognosis and the potential mediators involved. **Methods**. Seventy patients with acute anterior circulation ischemic stroke were included. Dietary patterns were evaluated using the MEDAS scale, a food-frequency questionnaire, and a 24 h recall. Circulating biomarkers including insulin resistance (HOMA index), adipokines (resistin, adiponectin, leptin), choline pathway metabolites (TMAO, betaine, choline), and endothelial progenitor cells (EPCs) were measured. Early neurological improvement (ENI) at 24 h, final infarct volume, and functional outcome at 3 months were assessed. **Results**. Adherence to MD and olive oil consumption were associated with a lower prevalence of diabetes and atherothrombotic stroke, and with lower levels of fasting glycemia, hemoglobinA1C, insulin resistance, and TMAO levels. Monounsaturated fatty acids and oleic acid consumption correlated with lower resistin levels, while olive oil consumption was significantly associated with EPC mobilization. Multivariate analysis showed that higher MD adherence was independently associated with ENI and good functional prognosis at 3 months. EPC mobilization, lower HOMA levels, and lower resistin levels were associated with ENI, a smaller infarct volume, and good functional outcome. **Conclusions**. MD was associated with better prognosis after ischemic stroke, potentially mediated by lower insulin resistance, increased EPC mobilization, and lower resistin levels, among other factors.

## 1. Introduction

Stroke is one of the leading causes of mortality and dependency in adults. Despite the development of effective treatments in the acute phase of ischemic stroke (IS), many patients do not achieve a favorable prognosis. Additionally, once the infarction is established, there is no therapy that promotes the recovery of damaged tissue. In this context, research on preconditioning factors that act before stroke, promoting neuroprotective and neurorepair effects after IS, is of great importance.

Healthy lifestyle habits, such as adherence to a Mediterranean diet (MD), have been associated with a lower incidence of vascular diseases and lower mortality due to vascular diseases [1]. Also, the MD has been associated with lower stroke incidence in a clinical trial [2] and in several population-based studies [3,4,5]. The MD is a typical dietary pattern of countries located around the Mediterranean Sea, characterized by a high consumption of plant-based foods as legumes, the use of olive oil as the main source of fat, a moderate/high intake of fish and shellfish, low intake of red meat, and the occasional consumption of alcohol, in the form of wine, during meals [6,7].

Beyond the role of the MD in the primary and secondary prevention of stroke, this dietary pattern and some of its components have also been associated with stroke prognosis. Previous clinical studies have pointed to an association of pre-stroke adherence to the MD and olive oil consumption with lower stroke severity at admission, with a lower atherosclerotic etiology of stroke [8,9], and with better functional prognosis at discharge [9] and at 3 months after suffering the ischemic insult [10]. The potential neuroprotective and neuroreparative effects of the MD have been investigated in animal models to elucidate the involved molecular mechanisms. These mechanisms are, among others, the anti-inflammatory and antithrombotic effects of the MD, the boosting of neovascularization, the optimization of endothelial and neuronal function, and the facilitation of neurogenesis [11,12].

The nutritional components of the MD with potential neuroprotective effects are multiple, including polyphenols from extra-virgin olive oil (EVOO) [11,13] and monounsaturated (MUFA) and polyunsaturated (PUFA) fatty acids. MUFAs, including oleic acid obtained mainly from EVOO in the MD, have shown an association with better lipid profiles and with a lower incidence of vascular disease and mortality in previous studies [14,15]. Similarly, PUFAs, including eicosapentaenoic acid (EPA) and docosahexaenoic acid (DHA), have also been reported to be associated with a reduction in vascular events and mortality [16,17,18,19]. Also, the administration of PUFAs has been shown to be associated with a better prognosis in animal models of cerebral ischemia [20,21].

Regarding the molecular pathways involved in the neuroprotective effects of the MD, there are a series of intermediaries related to the MD, such as circulating endothelial progenitor cells (EPCs), that can favor a better endothelial function and promote angiogenesis [22,23,24,25]. Also, choline pathway metabolites are closely associated with certain specific dietary patterns. Their main source is phosphatidylcholine, which is very common in some foods, such as red meat. This compound is metabolized through the gut microbiota to choline, betaine, and finally trimethylamine N-oxide (TMAO). These choline metabolites have been related to the incidence of stroke in previous studies [26,27] and, recently, TMAO has been pointed out as a possible independent factor not only in the incidence but also in the severity and prognosis of ischemic stroke, with lower levels of TMAO being protective [28]. Finally, MD may also promote an anti-inflammatory environment mediated by the different expressions of cytokines and adipokines. Adipokines are secreted by adipose tissue, have pro- and anti-inflammatory effects, and have been implicated in the development of cardiovascular diseases and stroke [29]. Adiponectin is the most common among them and appears to have a protective effect in the development of vascular diseases [30,31,32]. On the other hand, resistin has been associated with pro-inflammatory effects and an increased risk of ischemic stroke [33,34] and atherogenesis [35], with described associations between this adipokine and unstable carotid plaques [36], as well as intracranial stenosis and associated vascular events [37].

Our aim was to assess the relationship between adherence to the Mediterranean diet (MD) prior to ischemic stroke (IS) and functional prognosis, as well as to investigate the dietary components and potential circulating biomarkers involved in the neuroprotection of MD.

## 2. Materials and Methods

### 2.1. Patients

We designed a single-center observational and prospective study that included consecutive patients with acute IS. Inclusion criteria were as follows: patients with IS due to large vessel occlusion in anterior circulation undergoing endovascular therapy; pre-stroke modified Rankin scale (mRS) 0–1; and initial admission at Acute Stroke Unit. All patients were managed according to the recommendations of national and international guidelines. Patients who, due to their clinical condition, were unable to complete the surveys and did not have a valid person to contact, as well as those who were transferred early to other centers, were excluded from the study. This project had the approval of our Ethics Committee (CEIM-PA 2020/014) and all patients or relatives signed their informed consent. Demographic, anthropometric, radiological, and clinical variables were collected using a ase Report Form designed for the study. Among these clinical variables, we included an assessment of metabolic syndrome using the NCEP ATP-III criteria [38] and anthropometric evaluations, including body mass index (BMI) and abdominal waist measurements.

### 2.2. Evaluation of Adherence to MD and Dietary Recall

An evaluation of the diet was conducted upon admission through an interview with the patient or a direct cohabiting family member (if the patient was unable to respond), during which various questionnaires were applied: one to evaluate adherence to the Mediterranean diet, a food-frequency consumption questionnaire (FFQ), and a 24 h recall, as will be described below.

For the evaluation of pre-stroke adherence to MD, the previously validated 14-item questionnaire (MEDAS) was used [2,39]. To facilitate data interpretation, the results of the questionnaire were categorized into two groups based on the median of our cohort: low adherence if <9 points and high adherence for ≥9 points. To enhance the information provided by this questionnaire, additional questions were included regarding the frequency of food consumption for each item. These questions allowed for a more detailed assessment of dietary habits by capturing the frequency of intake for specific food items, similar to the food-frequency questionnaires used in large observational studies [40]. By gathering data on the frequency of food consumption, we were able to estimate daily intake in terms of servings per day and grams per day. This questionnaire can be consulted in the Supplementary Material (Table S1).

Additionally, a 24 h dietary recall was conducted to assess the foods and beverages consumed by the participant on the day prior to admission (or on a typical day if the day prior was not typical). This recall was either completed by the participant or by a cohabitant and offered insights into the participant’s typical dietary intake. From the data collected, we were able to calculate the macronutrient and micronutrient composition of the diet using the IENVA nutritional calculator (https://calcdieta.ienva.org/?lang=es, accessed on 30 July 2024). Specifically, we evaluated the quantity of proteins, carbohydrates, and lipids, wherein we assessed polyunsaturated fatty acids (including eicosapentaenoic acid (EPA) and docosapentaenoic acid (DPA)) and monounsaturated fatty acids (such as oleic and linoleic acid, among others). This approach allowed for a thorough assessment of participants’ dietary habits and nutritional intake, thereby enriching the data obtained from the adherence questionnaire.

### 2.3. Radiological Variables

All patients underwent a follow-up CT scan within the first 24 h (±12) post stroke to determine the presence or absence of hemorrhagic transformation according to the Heidelberg classification, as well as the final infarct volume. The infarct volume was manually calculated using the ellipsoid formula AxBxC/2 [41] and the results were expressed in mL or cc.

### 2.4. Laboratory Testing, Circulating Inflammatory Markers and Potential Intermediaries

All patients underwent baseline blood tests upon admission, which included measurements of total cholesterol, HDL cholesterol, LDL cholesterol, triglycerides, glucose, and insulin levels, among others. Insulin resistance (IR) was determined using the Homeostatic Model Assessment of Insulin Resistance (HOMA-IR), as previously described: [glucose (mg/dL) × insulin (mUI/mL)/405] [42].

Additionally, we collected samples for the determination of inflammatory markers, adipokines, EPCs and choline pathway metabolites (CPM) (betaine, choline, and TMAO). At 7 days post-stroke, or at discharge if it occurred before 7 days, we obtained a new sample to determine EPCs and assess the mobilization of these cells. Below, we briefly describe the methods used to determine each of these intermediaries.

#### 2.4.1. Endothelial Progenitor Cells

The determination of EPCs was performed at the Flow Cytometry Unit of the Institute of Health Research of the Principality of Asturias (ISPA) according to previously described methods [43]. Samples were processed within 1–2 h of collection by a single investigator blinded to the patients’ clinical or radiological results. In summary, the expression of specific surface antigens of circulating EPCs was analyzed using direct flow cytometry. Cells were labeled with anti-CD45 conjugated with PE-Cy7, anti-CD34 conjugated with FITC, anti-KDR conjugated with PE, and anti-CD133 conjugated with APC. Before lysing the erythrocytes with FACS lysing solution and without washing the samples, they were analyzed on an FACSAria IIu flow cytometer (BD, Franklin Lakes, NJ, USA). Cells with triple staining CD133+/CD34+/KDR+ in the mononuclear cell fraction were considered EPCs. Cell counts were always expressed per 10^6^ events. The “increase” in EPCs was determined when levels at 7 days/at discharge were higher than baseline levels.

#### 2.4.2. Choline Pathway Metabolites

Simultaneous metabolite quantification (choline, betaine, TMAO) was conducted via two-dimensional liquid chromatography and isotope dilution tandem mass spectrometry at the University of Oviedo. An Agilent 1290 Infinity II 2D-HPLC system with an Agilent 6460 triple quadrupole mass spectrometer (Agilent, Santa Clara, CA, USA) was employed, controlled by OpenLAB CDS ChemStation Edition C.01.07 SR3 and Agilent MassHUnter Workstation Software version B.08.02. Sample preparation involved mixing serum with isotopically labeled standards, protein precipitation with methanol, centrifugation, evaporation, reconstitution, and filtration. Chromatographic separation was achieved using ZORBAX Eclipse Plus C18 column (Agilent, Santa Clara, CA, USA) in the first dimension and Zorbax 300-SCX column (Agilent, Santa Clara, CA, USA) in the second, with positive ion electrospray ionization in MRM mode. MRM transitions, conditions of the ionization source, and collision energies were optimized for each metabolite.

#### 2.4.3. Adipokines

Adiponectin, leptin, and resistin levels were assessed using enzyme-linked immunosorbent assay (ELISA) kits from BioVendor R&D (Karàsek, Czech Republic). The assays were conducted at the Biochemistry Laboratory of the Central University Hospital of Asturias following the manufacturer’s instructions on a Triturus automated system (Grifols, Barcelona).

### 2.5. Prognostic Clinical Variables

Stroke severity at admission was assessed using the NIHSS scale [44]. Early neurological improvement (ENI) was determined as a reduction of 4 points or more on the NIHSS at 24 h after onset, and early dramatic neurological improvement (END) as a decrease of 10 points or more or a score of 0–1 at 24 h [45,46].

At 3 months, through presential visits in most cases or via telephone when not possible, functional prognosis was assessed using the modified Rankin Scale (mRS). Patients were categorized as having “good functional prognosis” when the mRS was 0–2 and as having “excellent functional prognosis” when they achieved values of 0 or 1 on the mRS at 3 months.

### 2.6. Statystical Analysis

The statistical analysis was carried out with the statistical package SPSS 24.0 (SPSS Inc., Chicago, IL, USA) for IOS. Categorical variables were presented as absolute values and percentages and continuous variables as mean (standard deviation (SD)) or [interquartile range] depending on normal distribution. Bivariate comparison between groups was conducted using the Chi-square test for categorical variables, Student’s *t* test for normally distributed quantitative variables, and Mann–Whitney U test for quantitative variables not following a normal distribution. The Pearson correlation coefficient (r) was used to establish a correlation between quantitative variables following a normal distribution, whereas the Spearman correlation coefficient (sp) was used for those not following a normal distribution. Significant associations were considered when *p* < 0.05. Multivariate analyses were performed, taking into account the usual variables associated with the studied prognostic factors and those that showed significant associations in univariate analyses. Results were expressed as adjusted odds ratios with corresponding 95% confidence intervals.

## 3. Results

### 3.1. General Description of the Sample

#### 3.1.1. Clinical and Diet Variables

Between 11 January 2021 and 14 June 2022, a total of 70 patients meeting the inclusion criteria and willing to participate were included. The mean age was 74.7 years (±10.7 SD), with 60% being males. The cutoff point we defined to determine whether a patient had good or poor adherence to the MD was 9 points on the MEDAS scale, coincident with the median of our sample. Thus, 52.9% of the sample had good adherence (MEDAS ≥ 9). Among patients with low adherence to MD, there was a higher prevalence of diabetes mellitus (DM) diagnosis (48.5% vs. 18.9%, *p* = 0.009) and significantly higher HbA1 levels, and a trend towards lower HDL cholesterol and higher fasting glucose levels. Additionally, tandem occlusions and atherothrombotic stroke were more frequent in the low adherence group (24.2% vs. 5.4%, *p* = 0.038; 18.2% vs. 2.7%, *p* = 0.046, respectively). We did not find significant differences in the rest of the analyzed characteristics, as shown in Table 1.

The daily consumption frequencies of the main foods in the Mediterranean diet among patients with low and high adherence can be found in Supplementary Table S2. Patients in the group with high adherence to the MD consumed more grams of olive oil, vegetables, nuts, red wine, poultry (chicken), and Sofrito, as well as fewer grams of commercial pastries. An analysis of the FFQ results revealed a significantly lower consumption of olive oil (g/day) in diabetic patients (30 g/day [30–60] vs. 60 g/day [45–60], *p* < 0.01) and in patients with atherothrombotic stroke (30 g/day [7.5–60] vs. 60 g/day [30–60], *p* = 0.06). Furthermore, a significant negative correlation was found between olive oil consumption and HbA1c levels (r = −0.28, *p* = 0.03).

In the analysis of 24 h dietary recall, the consumption of MUFAs, oleic acid and EPA, typical components of the MD, correlated positively with HDL–cholesterol levels (r = 0.45, r = 0.47, and r = 0.34, respectively; *p* < 0.01 in all cases). The results regarding the consumption of some macro- and micronutrients in the groups with high and low adherence to the MD can be found in Table S3 of the Supplementary Material.

#### 3.1.2. Circulating Biomarkers and Dietary Variables

Regarding the analyzed biomarkers, the group with higher adherence to MD had lower insulin resistance as determined by the HOMA index, although these differences were not statistically significant (Table 2). Regarding the components of the MD, we found a negative correlation between the HOMA index and olive oil consumption, as reported in the FFQ (r = −0.37, *p* = 0.003). In a similar way, a significant negative correlation was observed between the HOMA index and the consumption of MUFAs (−0.38, 0.006), and PUFAs (−0.36, *p* = 0.009). Specifically, significant correlations between the HOMA index and the consumption of certain MUFAs/PUFAs were found with oleic acid (−0.39, *p* = 0.004), linoleic acid (−0.33, *p* = 0.017), and DHA (−0.29, *p* = 0.034) in the 24 h dietary recall.

Among the circulating choline pathway metabolites, significantly lower TMAO levels were found in patients with high adherence to the MD (0.19 [0.11–0.28] vs. 0.25 [0.18–0.43], *p* < 0.05), Table 2. Regarding specific foods in the FFQ, we observed an inverse correlation between vegetable (r = −0.26, *p* = 0.03) and legume (r = −0.24, *p* = 0.04) consumption and TMAO circulating levels.

Among the analyzed adipokines, resistin levels were significantly higher in obese patients (10.5 [7.57–13.6] ng/mL in obese vs. 8.2 [6.09–10.97] in non-obese, *p* = 0.05), and were negatively correlated with MUFA consumption (r = −0.34, *p* = 0.008), and specifically with oleic acid consumption (−0.31, *p* = 0.017). Adiponectin levels were significantly lower in patients with atherothrombotic stroke (4.76 [3.76–12] vs. 11.35 [8.86–14.7] microg/nL, *p* = 0.02) and were positively correlated with MUFA consumption (r = 0.35, *p* = 0.005) and PUFA consumption (r = 0.29, *p* = 0.025), and specifically linoleic acid (r = 0.25, *p* = 0.049), EPA (r = 0.38, *p* = 0.003), and DHA (r = 0.27, *p* = 0.036). Leptin levels were significantly higher in obese patients but were not related to specific food consumption or adherence to the MD.

The median baseline EPC count was 30/10^6^ events [10–97.7], with an increase (mobilization) observed at 7 days/discharge in 66% of participants (median increase 35.5 cells/10^6^ events [17.4–93.2]). Those mobilizing EPCs consumed more olive oil (60 [30–60] g/day vs. 37.5 [30–60] g/day, *p* = 0.04) and had a lower IR as determined by the HOMA index (2.89 [1.89–5.12] vs. 6.01 [292–11.69], *p* = 0.027).

Table 2 summarizes the main results regarding biomarkers between patients with low and high adherence to MD.

### 3.2. Mediterranean Diet and Prognosis

Table 3 summarizes the main prognostic variables in the groups with different adherence to the MD. Patients with high adherence to MD experienced greater early neurological improvement (97.3% vs. 78.8%, *p* = 0.03) and a trend towards better functional prognosis at 3 months (mRS 0−2) (81.8% vs. 60.6%, *p* = 0.06). Among the foods assessed in the FFQ, the consumption of commercial pastries was significantly lower among those experiencing greater early neurological improvement (7 [0–50] g/day vs. 100 [62.5–100] g/day, *p* = 0.001) and in those with a better functional prognosis (mRS 0−2) at 3 months (7 [0–50] g/day vs. 100 [9–100] g/day, *p* = 0.038). Vegetable consumption, on the other hand, was significantly higher in those with excellent prognosis (mRS 0−1) at 3 months (400 [100–400] g/day vs. 200 [100–400] g/day, *p* = 0.034). In the multivariate analysis adjusted for significant cofactors in previous univariate analyses (Table 4 and Table 5), good adherence to the MD behaved as an independent predictor of early neurological improvement, with an OR of 11.98 (95% CI [1.1–130.32], *p* = 0.04), and of good functional prognosis (mRS 0−2) at 3 months, with an OR of 4.88 (95% CI [1.26–18.93], *p* = 0.02).

### 3.3. Potential Biomarkers Involved in Prognosis

Insulin resistance (IR) was associated with many of the studied prognostic variables, with HOMA levels beign significantly lower among those patients with greater early neurological improvement (12 [7–19.7] vs. 19 [13.23–45], *p* = 0.03) and also those with greater early dramatic neurological improvement (3 [1.89–6.24] vs. 4.75 [3.56–8.31], *p* = 0.024). Additionally, HOMA index positively correlated with final infarct volume (r = 0.67, *p* < 0.01).

Resistin levels were significantly lower in patients with good functional prognosis (mRS 0–2) at 3 months (7.96 [6.07–11.15] vs. 9.45 [8.07–14.35] ng/mL, *p* = 0.04).

We found a negative correlation between baseline EPC count and final infarct volume (r = −0.25, *p* = 0.036). EPC mobilization was more frequent among patients with early dramatic improvement (END) (84.6% in patients with END vs. 52.6% in patients without END, *p* = 0.019).

## 4. Discussion

In this observational study of patients with ischemic stroke treated with thrombectomy, we studied adherence to the Mediterranean diet (MD), intake of some specific nutrients, and different circulating biomarkers in relation to prognosis after stroke.

First of all, after multivariable logistic regression, higher adherence to the MD was independently associated with early neurological improvement and with good functional prognosis at three months. Regarding the potential mediators involved, we studied insulin resistance, circulating endothelial progenitor cells (CPEs), MUFAs and PUFAs intake, some circulating adipokines, and circulating choline-pathway metabolites. We present the principal findings below.

Insulin resistance (IR) is a known factor associated with worse stroke prognosis, in part due to the associated hyperglycemia and pro-inflammatory state, which leads to larger infarct volumes and the development of cerebral edema [47,48]. Since the MD can enhance insulin sensitivity, this could partially explain its neuroprotective and neurorestorative benefits. In our sample, IR was associated with larger infarcts and lower rates of ENI. In addition to being an important vascular risk factor [49], IR may also behave as an independent risk factor for poor outcome in non-diabetic stroke patients [50], and its prevention and treatment could be a fundamental therapeutic pillar. Regarding dietary habits, in our sample, IR was lower in patients with high adherence to the MD and was negatively correlated with olive oil and PUFA (linoleic and DHA) intake. Thus, we hypothesize that the benefits of a Mediterranean diet in terms of stroke prognosis may be mediated by the reduction of insulin resistance and its harmful effects.

Concerning circulating endothelial progenitor cells (EPCs), previous studies have shown positive effects on ischemic stroke, with EPCs being associated with lower infarct volumes and good functional prognosis [23,51,52]. In our study, EPC mobilization was associated with early neurological improvement and a lower infarct volume. Regarding dietary variables, EPC mobilization was associated with olive oil consumption and with a lower IR in our study. Prior studies have also reported associations between a Mediterranean diet and EPCs [22,24] and endothelial function [25]. Research in this line should be promoted to better study these associations.

As previously described, it would be the combination of several components comprising the MD, rather than any single food item, that would determine the observed benefits regarding vascular disease. Among these foods and their components are PUFAs and MUFAs. Based on this theory, animal models of ischemia were used to test the theory that the use of EPAs (an n-3 PUFA), either alone or in combination with other PUFAs, improved functional prognosis [20,21]. Similarly, it was shown that the use of ethyl icosapentate, a derivative of EPA, in patients at high vascular risk, reduced the incidence of vascular events [19]. On the other hand, hydroxytyrosol, one of the most common polyphenols in olive oil, has been associated in previous in vitro and in vivo studies with a reduction in inflammatory cytokines and the modulation of microglia in cerebral ischemia models [53,54]. Our results point to a better clinical outcome among patients with high adherence to the MD, which probably comes, at least in part, due to the components and typical foods of this type of diet, such as olive oil and its properties, as previously described [10]. Although the ultimate mechanisms are not clear, it is likely that part of the positive effect of these components is determined by their anti-inflammatory functions [55], which we support using some of the study’s findings. First, patients with low adherence to the MD presented with increased atherothrombotic stroke etiology. Atherosclerosis is a dynamic and complex disease, intimately related to inflammatory processes in its genesis, maintenance, and development [56]. Similarly, low levels of HDL cholesterol, beyond their implication in the development of atherosclerosis, have also been associated with a worse prognosis after suffering a stroke, in part due to their anti-inflammatory effects [57]. In our results, we found a positive correlation between EPA consumption and HDL cholesterol, so adherence to the MD and the consumption of PUFAs and specifically EPAs would favor a better lipid profile, with a lower inflammatory state.

The MD also appears to attenuate the inflammatory mechanisms involved in ischemic damage and recovery after stroke [58]. The results obtained in the analysis of adipokines could also support the theory that the anti-inflammatory mechanism is one of the main contributors. Adiponectin, a major adipokine with anti-inflammatory and anti-atherogenic functions [31,32], was significantly lower in patients with atherothrombotic stroke and positively correlated with MUFA and PUFA intake, reinforcing the available evidence regarding the effects of this adipokine [59,60]. On the other hand, we found a negative correlation between resistin levels, associated with inflammatory phenomena and vascular events [34,36,37], and the intake of MUFAs in general and oleic acid in particular. Also, lower resistin levels were observed in patients with better functional prognosis. In this regard, we provide new evidence that adherence to an MD and the components of this type of diet would favor a more favorable circulating adipokine profile, with higher levels of adiponectin and lower levels of resistin. Contrary to what is usually described, we found no relationship between the levels of these adipokines and obesity or waist circumference. Adipose tissue is considered to be an organ with endocrine functions, composed of different subtypes. White adipose tissue is one of them, predominant in adult humans and distributed mainly through subcutaneous tissue and visceral fat. This is the subtype that performs the main secretory function, and its dysfunction has been associated in recent studies with higher vascular risk [61]. In line with previous observations [62], we hypothesize that the Mediterranean diet would help to maintain adequate white adipose tissue function, which is fundamental for maintaining a balanced adipokine profile toward a less inflamed state.

Finally, we observed that patients with good adherence to the MD and with more vegetable and legume intake had lower circulating levels of TMAO, a choline pathway metabolite previously associated with cardiovascular events and stroke [28]. Although we did not find a direct relationship between TMAO and any of the analyzed prognostic variables in our small sample, research in this line deserves more attention.

Our study has limitations that we consider important to mention. First, it is an observational study and patients were included after suffering a stroke. Thus, all questionnaires about diet were applied retrospectively, so there may have been recall bias, which could have influenced the results. Moreover, the final sample size was smaller than that initially calculated due to recruitment difficulties due to coincidence with two COVID-19 pandemic waves. Finally, due to the observational design, the associations found in this study may not reflect direct effects and should be interpreted as hypothesis generators.

In summary, our study provides new evidence about the beneficial effect that the Mediterranean diet may have on the prognosis of ischemic stroke. Taken together, our results point out that MD may promote the interaction between different factors (insulin sensitivity, EPC mobilization, anti-inflammatory effects) that finally leads to a better functional recovery. Further studies are needed to clarify the exact underlying mechanisms behind those associations.

## 5. Conclusions

Greater adherence to the Mediterranean diet (MD) and the consumption of healthy fatty acids found in foods of this dietary pattern, especially in olive oil, were associated with better early neurological recovery after stroke and also with a better functional prognosis at 3 months. This association may be mediated, at least in part, by lower insulin resistance, the increased mobilization of EPCs, and the promotion of an anti-inflammatory state (i.e., lower levels of resistin).

## Figures and Tables

**Table 1:** Main differences between low/high adherence to the Mediterranean diet (MD).

	Global (n = 70)	Low Adherence to MD (n = 33)	High Adherence to MD (n = 37)	*p*
Age, years	74.7 ± 10.7	74.9 ± 12.5	73.9 ± 8.4	0.56
Gender (female)	28 (40)	14 (42.4)	14 (37.8)	0.69
Smokers	33 (48.6)	19 (57.6)	14 (40)	0.34
Alcohol intake	5 (7.1)	1 (3)	4 (10.8)	0.36
Dyslipidemia	40 (57.1)	21 (63.6)	19 (51.4)	0.30
Hypertension	44 (62.9)	20 (60.6)	24 (64.9)	0.71
Diabetes	23 (32.9)	16 (48.5)	7 (18.9)	**<0.01**
Atrial fibrillation	46 (65.7)	21 (63.7)	25 (53.5)	0.94
Clinical atherosclerosis ^1^	18 (25.7)	11 (33.3)	7 (18.9)	0.17
Metabolic syndrome	34 (48.6)	16 (48.5)	18 (48.6)	0.81
BMI	27.6 [25.2–30.1]	26.6 [24.7–28.9]	27.8 [24.2–30.6]	0.17
Obesity according to BMI	19 (27.1)	6 (18.2)	13 (35.1)	0.11
Waist circumference (cm)	101 [93–112]	97.9 [87–107]	99 [93.7–111.2]	0.11
Fasting glucose, mg/dL	109 [99.7–123.2]	111 [95–131]	105 [96.5–112.2]	**0.07**
Insulinemia, microU/mL	14.1 [8.7–21.8]	19 [9–23]	13.5 [9.6–17.5]	0.19
HbA1c	6 [6–6]	6 [6–7]	6 [5–6]	**0.03**
LDL–cholesterol, mg/dL	84 [62–112]	84 [65–96]	99.5 [63.5–123.7]	0.45
HDL–cholesterol, mg/dL	44 [35–56]	42 [32–52]	49 [39–59.5]	**0.07**
Triglycerides, mg/dL	95 [75.5–133]	98 [80–132]	91.5 [74–138.5]	0.85
Occlusion site				0.73
TICA	11 (15.7)	5 (15.2)	6 (16.2)	
M1	37 (52.9)	19 (57.6)	18 (48.6)	
M2	22 (31.4)	9 (27.3)	13 (35.1)	
Tandem occlusions (%)	10 (14.3)	8 (24.2)	2 (5.4)	**0.04**
ASPECTS	8 [7–9]	8 [7–9]	7.5 [6–9]	0.35
TOAST ethiology				
Aterothrombotic	7 (10)	6 (18.2)	1 (2.7)	**0.04**
Cardioembolic	49 (70)	21 (63.6)	28 (75.7)	
Unusual	3 (4.3)	1 (3)	2 (5.4)	
Undetermined	11 (15.7)	5 (15.2)	6 (16.2)	

^1^ Clinical atherosclerosis includes prior ischemic heart disease, prior stroke and/or peripheral arteriopathy. BMI, body mass index; TICA, terminal internal carotid artery; M1, proximal middle cerebral artery; M2, distal middle cerebral artery; ASPECTS, Alberta stroke program early CT score. TOAST, Trial of ORG, 10,172 in acute stroke treatment. ***p*** in bold if statistically significant (<0.05).

**Table 2:** Biomarkers and bioactive compounds between low and high adherence.

	Global (n = 70)	Low Adherence (n = 33)	High Adherence (n = 37)	*p*
HOMA index	3.91 [1.97–6.63]	4.66 [2–7.1]	3.5 [1.96–5.1]	0.22
Baseline EPC, mill/events	30 [10–97.75]	22.5 [10–101.5]	45 [9–97.5]	0.61
EPC’s increase ^1^, %	32 (71.1)	17 (73.9)	15 (68.2)	0.67
Betaine, PPM	3.55 [2.77–4.44]	3.59 [2.77–4.84]	3.6 [3.14–4.27]	0.28
Choline, PPM	1.89 [1.63–2.34]	1.87 [1.54–2.49]	1.86 [1.63–2.15]	0.97
TMAO, PPM	0.21 [0.12–0.35]	0.25 [0.18–0.43]	0.19 [0.11–0.28]	**0.002**
Resistin, ng/mL	8.62 [6.72–11.85]	8.83 [6.08–11.5]	7.96 [6.73–9.83]	0.4
Leptin, ng/dL	13 [7.35–33.05]	12.95 [5.57–20.9]	15.5 [9.55–47.05]	0.15
Adiponectin, µg/mL	11.3 [7.5–14.6]	10.5 [5.53–12.45]	11.7 [9.4–15.35]	0.53

^1^ An EPC increase could only be estimated in 45 out of the total. Results are expressed as median [interquartile range]. ***p*** in bold if statistically significant (<0.05).

**Table 3:** Low/high adherence to MD and prognostic variables.

	Global (n = 70)	Low Adherence (n = 33)	High Adherence (n = 37)	*p*
NIHSS pre-treatment	14 [10–18]	13 [10–17]	14 [10–18]	0.48
ENI	62 (88.6)	26 (78.8)	36 (97.3)	**0.03**
END	44 (62.9)	18 (54.5)	26 (70.3)	0.17
Final infarct volume, mL	7.8 [1.3–25.2]	7.9 [1.5–23.9]	6.2 [1.2–25.6]	0.75
mRS 0–2 at 3 months	50 (71.4)	20 (60.6)	30 (81.8)	0.06
mRS 0–1 at 3 months	35 (50)	16 (48.5)	19 (51.4)	0.4

ENI: early neurological improvement; END: early neurological dramatical improvement; NIHSS, National Institute of Health Stroke Scale; mRS, modified Rankin scale. ***p*** in bold if statistically significant (<0.05).

**Table 4:** Multivariate regression analysis for early neurological improvement.

	OR (95% IC)	*p* Value
High adherence to MD	11.98 (1.1–130.32)	0.04
Age	0.88 (0.78–0.99)	0.04
Sex, women	17.73 (1.11–283.91)	0.04
Fasting glucose	0.97 (0.93–1)	0.11

**Table 5:** Multivariate regression analysis for good functional prognosis at 3 months (mRS 0–2).

	OR (95% IC)	*p* Value
High adherence to MD	4.88 (1.26–18.93)	0.02
Age	0.94 (0.88–0.99)	0.04
Sex, women	1.37 (0.4–4.67)	0.61
NIHSS pre-treatment	0.84 (0.74–0.96)	0.01

## Data Availability

The data presented in this study are available upon request from the corresponding author.

## Supplementary Materials

The following supporting information can be downloaded at: https://www.mdpi.com/article/10.3390/nu16183218/s1. Table S1: MEDAS and food-frequency questionnaire; Table S2: Daily consumption frequencies of the main foods in the Mediterranean diet among patients with low and high adherence to MD; Table S3: Results of 24-h diet recall among low and high adherence to MD.
