# Supplementary material for: Mediterranean Diet Prior to Ischemic Stroke and Potential Circulating Mediators of Favorable Outcomes
Source: Nutrients. 2024 Sep 23;16(18):3218. doi: 10.3390/nu16183218 (PMC11435288; doi:10.3390/nu16183218)
Supplement: Supplementary file 1 [file nutrients-16-03218-s001.zip › nutrients-3158784-supplementary.pdf]

SUPPLEMENTARY MATERIAL

Title: Mediterranean Diet prior to ischemic stroke and potential mediators of favorable outcomes.

Authors:

1. **Supplementary Data**
   No supplementary data are provided.

2. **Supplementary Tables**

Table S1: MEDAS and food-frequency questionnaire

Do you use olive oil as the main culinary fat?

No – 0 points   Yes – 1 point

How much olive oil do you consume on a typical day (including what you use for frying, meals outside the home, salads, etc.)? (1 serving = 1 tablespoon = 10 ml)

<4 servings – 0 points   ≥ 4 – 1 point

Provide an estimate of approximately how many tablespoons (per day):

On average, what would you say is your frequency of consumption for this food? (Please select the option you feel is most accurate.)

Never or almost never	Monthly	Weekly			Daily			
	1 – 3	1	2 – 4	5 – 6	1	2 – 3	4 – 6	6 +

How many vegetable servings do you consume per day? 1 serving: 200 g (consider side dishes as half a serving)

< 2 - 0 points   ≥ 2 – 1 point

Provide approximately how many (per day).

On average, what would you say is your frequency of consumption for this food? (Please select the option you feel is most accurate.)

Never or almost never	Monthly	Weekly			Daily			
	1 – 3	1	2 – 4	5 – 6	1	2 – 3	4 – 6	6 +

How many fruit units (including natural fruit juices) do you consume per day?

< 3 – 0 point   ≥ 3 or more – 1 point

Provide approximately how many (per day).

On average, what would you say is your frequency of consumption for this food? (Please select the option you feel is most accurate.)

Never or almost never	Monthly	Weekly			Daily			
	1 – 3	1	2 – 4	5 – 6	1	2 – 3	4 – 6	6 +

How many servings of red meat, hamburger, or meat products (ham, sausage, etc.) do you consume per day?

> 1 - 0 points ≤ 1 – 1 point

Provide approximately how many (per week).

On average, what would you say is your frequency of consumption for this food? (Please select the option you feel is most accurate.)

Never or almost never	Monthly	Weekly			Daily			
	1 – 3	1	2 – 4	5 – 6	1	2 – 3	4 – 6	6 +

How many servings of butter, margarine, or cream do you consume per day? (1 serving: 12 g)

> 1 - 0 points ≤ 1 – 1 point

Provide approximately how many (per week).

On average, what would you say is your frequency of consumption for this food? (Please select the option you feel is most accurate.)

Never or almost never	Monthly	Weekly			Daily			
	1 – 3	1	2 – 4	5 – 6	1	2 – 3	4 – 6	6 +

How many sweetened and/or carbonated beverages do you drink per day?

> 1 - 0 points ≤ 1 – 1 point

Provide approximately how many (per week).

On average, what would you say is your frequency of consumption for this food? (Please select the option you feel is most accurate.)

Never or almost never	Monthly	Weekly			Daily			
	1 – 3	1	2 – 4	5 – 6	1	2 – 3	4 – 6	6 +

How much wine do you drink per week? (glasses)

< 7 – 0 points ≥ 7– 1 point

Provide approximately how many glasses (per week).

On average, what would you say is your frequency of consumption for this food? (Please select the option you feel is most accurate.)

Never or almost never	Monthly	Weekly			Daily			
	1 – 3	1	2 – 4	5 – 6	1	2 – 3	4 – 6	6 +

How many servings of legumes do you consume per week? (1 serving: 150 g)

< 3 - 0 puntos ≥ 3 - 1 punto

Provide approximately how many (per week).

On average, what would you say is your frequency of consumption for this food? (Please select the option you feel is most accurate.)

Never or almost never	Monthly	Weekly			Daily			
	1 – 3	1	2 – 4	5 – 6	1	2 – 3	4 – 6	6 +

How many servings of fish or shellfish do you consume per week? (1 serving 100–150 g of fish or 4–5 units or 200 g of shellfish)

< 3 - 0 points ≥ 3 - 1 point

Provide approximately how many (per week).

On average, what would you say is your frequency of consumption for this food? (Please select the option you feel is most accurate.)

Never or almost never	Monthly	Weekly			Daily			
	1 – 3	1	2 – 4	5 – 6	1	2 – 3	4 – 6	6 +

How many times per week do you consume commercial sweets or pastries (not homemade), such as cakes, cookies, biscuits, or custard?

≥ 2 - 0 points < 2 – 1 point

Provide approximately how many (per week).

On average, what would you say is your frequency of consumption for this food? (Please select the option you feel is most accurate.)

Never or almost never	Monthly	Weekly			Daily			
	1 – 3	1	2 – 4	5 – 6	1	2 – 3	4 – 6	6 +

How many servings of nuts (including peanuts) do you consume per week? (1 serving 30 g)

< 3 - 0 points ≥ 3 - 1 point

Provide approximately how many (per week).

On average, what would you say is your frequency of consumption for this food? (Please select the option you feel is most accurate.)

Never or almost never	Monthly	Weekly			Daily			
	1 – 3	1	2 – 4	5 – 6	1	2 – 3	4 – 6	6 +

Do you preferentially consume chicken, turkey, or rabbit meat instead of veal, pork, hamburger, or sausage?

No - 0 points Yes - 1 point

Provide approximately how many (per week).

On average, what would you say is your frequency of consumption for this food? (Please select the option you feel is most accurate.)

Never or almost never	Monthly	Weekly			Daily			
	1 – 3	1	2 – 4	5 – 6	1	2 – 3	4 – 6	6 +

How many times per week do you consume vegetables, pasta, rice, or other dishes seasoned with *sofrito* (sauce made with tomato and onion, leek or garlic, and simmered with olive oil)?

< 2 - 0 points ≥ 2 - 1 point

Provide approximately how many (per week).

On average, what would you say is your frequency of consumption for this food? (Please select the option you feel is most accurate.)

Never or almost never	Monthly	Weekly			Daily			
	1 – 3	1	2 – 4	5 – 6	1	2 – 3	4 – 6	6 +

Table S2: Daily consumption frequencies of the main foods in the Mediterranean diet among patients with low and high adherence to the MD.

	**Global (n=70)**	**Low adherence to MD (n=33)**	**High adherence to MD (n=37)**	**p**
Olive oil (g/day)	60 [30 – 60]	30 [30 – 60]	60 [60 – 60]	<0.01
Vegetable (g/day)	200 [100 – 400]	200 [100 - 400]	400 [100 - 400]	0.02
Fruit (g/day)	300 [150 – 450]	300 [150 – 450]	300 [150 – 450]	0.78
Red meat (g/day)	35.7 [8.9 – 53.6]	35.7 [8.9 – 62.5]	35.7 [17.8 – 53.6]	0.67
Butter (g/day)	0 [0 – 1]	0 [0 – 1.3]	0 [0 – 1.3]	0.47
Carbonated beverages (cc/day)	0 [0 – 14.3]	0 [0 – 14.2]	0 [0 – 7.1]	0.68
Wine (cc/day)	0 [0 – 32.1]	0 [0 – 3.5]	0 [0 – 100]	0.05
Legumes (g/day)	85.7 [57.1 – 114.3]	85.7 [57.1 – 85.7]	85.7 [57.1 – 114.3]	0.12
Fish (g/day)	35.7 [17.8 – 53.6]	35.7 [17.8 – 53.6]	35.7 [26.8 – 53.6]	0.36
Commercial sweets or pastries (g/day)	7 [0 – 62.5]	50 [3.5 – 100]	0 [0 – 14.5]	<0.01
Nuts (g/day)	2.1 [0 – 12.8]	0 [0 – 2.1]	8.6 [1 – 30]	<0.01
Chicken, turkey or rabbit meat (g/day)	35.7 [15.6 – 53.6]	17.8 [8.9 – 35.7]	35.7 [17.8 – 53.6]	<0.01
Sofrito (servings/day)	0.3 [0.1 – 0.3]	0.1 [0 – 0.3]	0.3 [0.2 – 0.6]	<0.01

Sofrito: sauce made with tomato, onion and garlic and simmered with olive oil. Data are expressed as median [interquartile range].

Table S3: Results of 24-hour diet recall among patients with low and high adherence to the MD.

	**Global (n=70)**	**Low adherence to MD (n=33)**	**High adherence to MD (n=37)**	**p**
Energy, kcal/day	1300.96 [1088.64 – 1733.38]	1263.99 [903.57 – 1588.45]	1237.66 [1139.89-1668.17]	0.35
Proteins, g/day	54.73 [40.52 – 71.98]	49.46 [33.61 – 68.2]	54.94 [45.85 – 72.09]	0.2
Carbohydrates, g/day	115.31 [89.23 – 171.68]	110.56 [81.99 – 155.98]	114.45 [98.63 – 145.38]	0.59
Lipids, g/day	65.09 [51.58 – 92.35]	65.09 [49.94 – 83.66]	57.4 [50.95 – 94.32]	0.67
MUFA, g/day	30.12 [22.88 – 39.51]	28.49 [20.46 – 36.59]	31.5 [23.28 – 46.9]	0.23
Oleic acid, g/day	28.22 [21.37 – 38.01]	27.02 [18.78 – 34.23]	29.76 [22.01 – 43.15]	0.22
Linoleic acid, g/day	6.1 [4.23 – 11.52]	5.86 [3.57 – 9.42]	6.51 [4.63 – 12.85]	0.48
PUFA, g/day	7.54 [5.05 – 12.92]	7.2 [4.52 – 10.99]	7.58 [5.61 – 14.09]	0.47
EPA, g/day	0 [0 – 0.01]	0	0 [0-0.05]	0.18
DHA, g/day	0 [0 – 0.06]	0	0.01 [0-1]	0.28
